# Comparative Investigation of Red Wine Concentrates as Multifunctional Skin-Protective Agents

**DOI:** 10.3390/pharmaceutics18081034

**Published:** 2026-08-20

**Authors:** Gorana Ilić, Ivana Beara, Ljiljana Milovanović, Aleksandra Jovanović, Dragana Dekanski, Andrea Pirković

**Affiliations:** 1 Institute for the Application of Nuclear Energy—INEP, University of Belgrade, 11000 Belgrade, Serbia; ajovanovic@inep.co.rs (A.J.); dragana.dekanski@inep.co.rs (D.D.); andrea.pirkovic@inep.co.rs (A.P.); 2 Department of Chemistry, Biochemistry and Environmental Protection, Faculty of Sciences, University of Novi Sad, 21000 Novi Sad, Serbia; ivana.beara@dh.uns.ac.rs (I.B.); ljiljana.milovanovic@dh.uns.ac.rs (L.M.)

**Keywords:** *Vitis vinifera*, wine concentrate, skin, HaCaT, anti-tyrosinase, anti-elastase, anti-collagenase, antioxidant, anti-inflammatory, wound healing

## Abstract

**Background/Objectives**: *Vitis vinifera* is a rich source of polyphenolic compounds that are known for their benefits on skin health, including antioxidant, anti-inflammatory, and photoprotective effects. Red wines have shown a positive impact on cardiovascular disease, diabetes, and cancer prevention; however, evidence regarding their effects on skin biology remains scarce. This study evaluated red wine concentrates obtained from Cabernet Sauvignon and Merlot varieties, selected based on their previously demonstrated biological activities, to identify the samples with the highest potential for skin health applications by assessing their inhibitory activity against skin-related enzymes (tyrosinase, elastase, and collagenase), along with their effects on HaCaT cells, including cytotoxic, antioxidant, anti-inflammatory, and wound healing activities. **Methods**: Enzyme inhibition studies were based on spectrophotometric methods; the MTT and CV assays were carried out to test the cytotoxicity, and the DCFH-DA assay was employed to evaluate the antioxidant activity of the wine concentrates; wound healing potential was examined by the cell scratch assay, and anti-inflammatory activity was investigated using ELISA and qPCR analyses. PCA of the samples and their biological activity was performed as well. **Results**: Examined red wine concentrates exhibited significant enzyme inhibitory activity and showed no cytotoxicity on HaCaT cells up to the concentration of 50 μg/mL; the samples stimulated cell migration, inhibited intracellular ROS production in AAPH-stressed keratinocytes, and selectively modulated TNF-α-induced inflammatory responses, reducing IL-8 secretion while enhancing certain cytokine transcripts, underscoring the complexity of their bioactive effects. **Conclusions**: Red wine concentrates showed significant potential as skin-beneficial agents, demonstrating strong antiaging, marked cellular antioxidant effects and wound healing activity when applied at non-cytotoxic concentrations. Multivariate analysis revealed distinct bioactivity profiles among the wine concentrates.

## 1. Introduction

Grapevine (*Vitis vinifera* L., Vitaceae) contains a broad range of bioactive polyphenols, including flavonoids, anthocyanins, and stilbenes, with resveratrol being one of the most extensively studied compounds due to its beneficial effects on skin health [1]. These bioactive compounds exhibit strong antioxidant and anti-inflammatory activities, contributing to protection against oxidative stress, premature skin aging, and ultraviolet (UV)-induced cellular damage [2,3].

The sensory properties and health-promoting potential of *V. vinifera* grapes used in winemaking largely depend on their polyphenolic profile. During wine production, some of the polyphenolic compounds are degraded, but the main part remains. The polyphenolic profile of wines depends on various factors, including wine variety, viticulture practice, vineyard location, and winemaking conditions. Nevertheless, the most abundant polyphenols present in wines are phenolic acids, flavonoids, stilbenes, and tannins [4,5]. Red wines, in particular, have shown benefits on human health and a positive impact on cardiovascular disease, diabetes, and cancer prevention, as well as on gut microbiota and oral health [6]. Topical administration of several red wine-derived polyphenols demonstrated significant anticancer effects in a two-stage CD-1 mouse skin cancer model, with all tested compounds reducing tumor formation. The study also showed that *trans*-resveratrol, as the main bioactive component, is absorbed much more efficiently via topical application than after oral consumption [7].

Skin is a multifunctional organ that serves as the main barrier, protecting the organism from UV radiation, pathogens, pollution, and dehydration. The epidermis is the outermost layer of the skin, and it consists of keratinocytes, melanocytes, and Langerhans cells, with keratinocytes being the most prevalent cell type. Their main function is to establish a physical barrier to prevent skin damage. The dermis, as the middle layer of the skin, is mainly composed of dense extracellular matrix (ECM), derived from fibroblasts, the predominant cell type. The main ECM components are collagens, elastin fibers, proteoglycans, and hyaluronic acid, while less abundant are proteases, protease inhibitors, and cytokines [8]. Elastin is responsible for the skin’s elasticity, and collagen provides structural strength and support [9]. Proteases have a crucial role in ECM remodeling [8]. Elastase and collagenase are enzymes that are responsible for the degradation of elastin and collagen, respectively [9]. They are secreted in response to cytokines, oxidative stress, and UV radiation. The breakdown of elastin and collagen leads to skin aging and wrinkle formation [10].

Skin aging is a biological process induced by intrinsic and extrinsic factors, including genetic and hormonal influences, as well as UV radiation, pollution, and chemical agents. Skin aging is primarily characterized by wrinkle formation, dryness, and reduced elasticity and firmness [11]. Additionally, excessive production of reactive oxygen species (ROS) may cause skin hyperpigmentation. ROS stimulate tyrosinase activity, which catalyzes the conversion of L-tyrosine to melanin [12]. Melanin acts as a photoprotective agent against UV radiation; however, its overaccumulation results in hyperpigmented spots in the skin [13].

Previous studies evaluating wine- or grape-derived polyphenols in keratinocytes and other cell models relevant to skin biology have reported several benefits. For example, anthocyanins isolated from red wine were shown to improve keratinocyte barrier integrity [14]. In addition, comparative analyses of Merlot, Syrah, Tannat, and other *V. vinifera* cultivars demonstrated substantial differences in polyphenol profiles and cellular antioxidant/anti-inflammatory activities in macrophages, suggesting that biological effects may vary significantly among varieties [15,16]. However, direct comparative studies evaluating multiple red wine varieties specifically in skin cell systems, such as keratinocytes, wound healing models, or intracellular antioxidant assays, remain scarce. The majority of available evidence remains indirect and is not derived from specifically designed dermatological in vitro studies. In this study, the aim was to investigate and compare the potential of wine concentrates obtained from four varietal red wines (Serbian Cabernet Sauvignon and North Macedonian, Serbian, and Italian Merlot) as a source of skin-friendly agents that promote the protection and restoration of skin function. Our objective was to determine whether varietal and regional differences in polyphenol composition translate into distinct dermatological bioactivities. To address this, we conducted comparative analyses in keratinocytes and complementary biochemical assays in vitro. These included biochemical enzyme inhibition assays relevant for skin biology (anti-collagenase, anti-elastase, and anti-tyrosinase activities), as well as in vitro biological assays on human keratinocytes (HaCaT cell line) to identify safe, non-cytotoxic concentration ranges and investigate the antioxidant, anti-inflammatory, and wound healing potential of these wine concentrates. Finally, to comparatively assess the skin-beneficial potential of red wine concentrates, principal component analysis was performed to identify associations between wine varieties and their biological activities.

## 2. Materials and Methods

### 2.1. Preparation of Wine Concentrates

The study included four varietal wines produced in wineries in the Republic of Serbia, North Macedonia, and Italy, and found on the Serbian market in 2022 (W1—Serbian Cabernet Sauvignon, W2—North Macedonian Merlot, W3—Italian Merlot, and W4—Serbian Merlot). Wine samples were evaporated in vacuo at 45 °C. Dry residue was reconstituted in dimethyl sulfoxide (DMSO, Thermo Fisher Scientific, Waltham, MA, USA) to a final concentration of 200 mg of dry residue/mL (mg d.r./mL). All extracts were dissolved in DMSO immediately after preparation, aliquoted, and stored at −20 °C to prevent degradation until use in analyses.

The four wine concentrates included in the present study were selected based on the results of our previous phytochemical and biological investigations, in which these Cabernet Sauvignon and Merlot wines exhibited distinct polyphenolic profiles together with promising antioxidant, anti-inflammatory, and hypoglycemic activities [16,17,18]. Specifically, the contents of total polyphenols, flavonoids, tannins, and anthocyanins were evaluated using spectrophotometric methods, while targeted polyphenols were determined by HPLC analysis, as previously described in Majkić et al. [17]. The previously demonstrated biological activities of the selected wine concentrates made them suitable candidates for the evaluation of skin-related bioactivities.

### 2.2. Assessment of Skin-Related Enzyme Inhibition

#### 2.2.1. Inhibition of Tyrosinase

Tyrosinase inhibitory activity was measured spectrophotometrically, based on the method described by Majkić et al. [16]. In brief, 80 μL of 0.1 M potassium phosphate buffer (pH 6.8, Sigma-Aldrich, St. Louis, MO, USA) and 80 μL of tyrosinase from mushroom (0.02 mg/mL, Sigma-Aldrich, St. Louis, MO, USA; dissolved in 0.1 M potassium phosphate buffer, pH 6.8) were mixed with 20 μL of the tested samples and incubated for 15 min at 37 °C. To initiate the reaction, 2.5 mM 3,4-dihydroxy-L-phenylalanine (L-DOPA, Sigma-Aldrich, St. Louis, MO, USA) was added. The final concentration of DMSO (Thermo Fisher Scientific, Waltham, MA, USA) was 0.5% (*v*/*v*), and the corresponding vehicle controls contained the same final concentration of DMSO to control for solvent-related effects. The blank probe contained buffer instead of the substrate. After 25 min of incubation at 37 °C, the absorbance was measured at 475 nm (Multiskan Spectrum v1.2, Thermo Fisher Scientific, Waltham, MA, USA). Kojic acid (Sigma-Aldrich, St. Louis, MO, USA) was used as a positive control. The results were expressed as µg kojic acid equivalents per mg of dry weight (µg eq kojic acid/mg dw).

#### 2.2.2. Inhibition of Elastase

The assay of elastase inhibitory activity was based on a spectrophotometric method described by Sadeghi-Kaji et al. [19], with some modifications. Briefly, 120 μL of 0.1 M Tris-HCl buffer (pH 8, Sigma-Aldrich, St. Louis, MO, USA) and 25 μL of elastase from porcine pancreas (0.25 U/mL, Sigma-Aldrich, St. Louis, MO, USA; dissolved in Tris-HCl buffer) were mixed with 30 μL of the tested samples (100 mg/mL) and incubated for 20 min at room temperature. To eliminate potential solvent-related effects, the vehicle control contained the same final concentration of DMSO (0.75%, *v*/*v*, Thermo Fisher Scientific, Waltham, MA, USA) as the tested samples. The blank probe contained buffer instead of the substrate. The reaction was initiated by adding 5 mM N-Succinyl-Ala-Ala-Ala-*p*-nitroanilide (SANA, Sigma-Aldrich, St. Louis, MO, USA). After 20 min of incubation at room temperature, the absorbance was measured at 405 nm (Multiskan Spectrum v1.2, Thermo Fisher Scientific, Waltham, MA, USA). (-)-Epigallocatechin gallate (EGCG, Thermo Fisher Scientific, Waltham, MA, USA) was used as a positive control. The results were expressed as µg EGCG equivalents per mg of dry weight (µg eq EGCG/mg dw).

#### 2.2.3. Inhibition of Collagenase

The assay of collagenase inhibitory activity was based on the spectrophotometric method reported by Bahadır Acıkara et al. [9], with some modifications. Tricine buffer (Sigma-Aldrich, St. Louis, MO, USA) was used for this assay. Collagenase from *Clostridium histolyticum* (Sigma-Aldrich, St. Louis, MO, USA) was added to the Tricine buffer (50 mM, pH 7.5, 400 mM NaCl, and 10 mM CaCl_2_) to reach a concentration of 1 mg/mL. The substrate N-[3-(2-furyl)acryloyl]-Leu-Gly-Pro-Ala (FALGPA, Sigma-Aldrich, St. Louis, MO, USA) was added to the Tricine buffer to achieve a concentration of 0.75 mM. In each case, the mixture included 25 μL of the Tricine buffer, 50 μL of FALGPA, 25 μL of the enzyme, and 25 μL of the sample solution (2.5 mg/mL). The vehicle control was prepared with the same final concentration of DMSO (0.75%, *v*/*v*, Thermo Fisher Scientific, Waltham, MA, USA) as the tested samples to account for any solvent-related effects. Each sample was incubated with the enzyme in buffer for 20 min, after which the substrate was added to initiate the reaction. After adding the substrate, the absorbance was measured at 330 nm (Multiskan Spectrum v1.2, Thermo Fisher Scientific, Waltham, MA, USA). EGCG (0.7 mg/mL) was used as a positive control. The results were expressed as µg EGCG equivalents per mg of dry weight (µg eq EGCG/mg dw).

### 2.3. Cell Culture

The spontaneously immortalized human keratinocyte (HaCaT) cell line (a generous gift from Dr. Milica Pešić, Institute for Biological Research “Siniša Stanković”, University of Belgrade, Serbia) was used for cell-based assays. It was cultured in Dulbecco’s Modified Eagle’s Medium/Nutrient Mixture F-12 Ham (DMEM-F12, Biowest, Nuaillé, France), with 10% heat-inactivated fetal bovine serum (FBS, Biowest, Nuaillé, France) and 1% antibiotic/antimycotic solution (Capricorn Scientific GmbH, Ebsdorfergrund, Germany). The cells were maintained at 37 °C, under a humidified atmosphere and 5% CO_2_.

### 2.4. Cell Viability Assays

For the cytotoxicity assessment, two complementary assays were employed, MTT and crystal violet (CV) staining, in order to validate cell viability using two independent approaches. The MTT assay reflects the metabolic activity of viable cells, whereas the CV assay estimates the number of viable adherent cells by staining intracellular proteins and DNA of cells that remain attached to the culture surface [20]. In accordance with commonly accepted criteria, a treatment is generally considered non-cytotoxic when cell viability remains above 90% (i.e., less than a 10% reduction compared with the untreated control). Under standard regulatory guidelines, like ISO 10993-5:2009 [21], a material is considered non-cytotoxic if cell viability remains at or above 70%, while a cell viability result of above 90% reflects exceptional cytocompatibility, as well as absence of cytotoxic effect.

#### 2.4.1. MTT Assay

HaCaT cells were seeded in a 96-well plate at 2 × 10^4^ cells/well, in a final volume of 100 μL per well. After 24 h, the cells were treated with wine concentrates in a range of concentrations, from 12.5 μg/mL to 400 μg/mL (serial dilutions in complete DMEM-F12 medium), and incubated for another 24 h at 37 °C. Complete DMEM-F12 medium with a matching concentration of DMSO (0.2% *v*/*v*), to account for vehicle-induced cytotoxicity, was used as a control. Next, an MTT reagent (0.5 mg/mL, SERVA Electrophoresis GmbH, Heidelberg, Germany) was added to the cells and incubated for 3 h at 37 °C for the reaction to occur. Newly formed formazan crystals were dissolved using 10% sodium dodecyl sulfate (SDS, Sigma-Aldrich, St. Louis, MO, USA) in 0.01 M HCl. Finally, after complete solubilization of the crystals, absorbance was measured at 570 nm using a microplate reader (BioTek Epoch Microplate Spectrophotometer, Agilent BioTek Instruments, Santa Clara, CA, USA). Data were expressed as a percentage of viability compared to the control (100%). Mean values were represented as bars, from at least three independent experiments performed in triplicate (*n* = 9).

#### 2.4.2. Crystal Violet (CV) Assay

HaCaT cells were seeded in a 96-well plate at 2 × 10^4^ cells/well, in a final volume of 100 μL per well. After 24 h, the cells were treated with wine concentrates in a range of concentrations, from 12.5 μg/mL to 400 μg/mL (serial dilutions in complete DMEM-F12 medium), and incubated for another 24 h at 37 °C. The corresponding vehicle control contained the same final concentration of DMSO as the treatment groups (0.2% *v*/*v*), thereby controlling for any potential solvent-related effects. After incubation of the cells with the treatment preparations, the medium was removed, and the cells were washed with phosphate-buffered saline (PBS), dried, and fixed with an ice-cold acetone:methanol (1:1) mixture. After removal of the fixative and complete drying of the cells, 50 µL of 0.05% CV dye was added to each well. After a 5 min staining period, the excess dye was removed by washing the plates three times with distilled water and drying in air. The integrated dye was dissolved by adding 0.1 M sodium citrate in 50% ethanol. The optical density was measured at 570 nm using a microplate reader (BioTek Epoch Microplate Spectrophotometer, Agilent BioTek Instruments, Santa Clara, CA, USA). The entire experiment was performed three times in triplicate (*n* = 9). Results were expressed as a percentage of control values obtained for untreated cells. The significance level was *p* < 0.05.

### 2.5. Measurement of Intracellular Reactive Oxygen Species (ROS) Production

Antioxidant potential of wine concentrates was determined by 2′,7′-dichlorofluorescein diacetate (DCFH-DA) assay, measuring the production of intracellular ROS. 2,2′-Azobisisobutyramidinium chloride (AAPH, Acros Organics, Morris Plains, NJ, USA) was used as an ROS inducer. HaCaT cells were seeded in a 96-well plate at 2 × 10^4^ cells/well, in a final volume of 100 μL per well. On the following day, the medium was discarded, and the cells were incubated with 5 μM DCFH-DA (CAS 4091-99-0, Merck Millipore Calbiochem, San Diego, CA, USA) in PBS containing 10% FBS for 45 min in the dark. After that, the cells were rinsed with PBS and co-incubated with samples (12.5, 25, and 50 μg/mL, i.e., non-toxic concentrations) and 30 mM AAPH for another 1 h. The AAPH concentration was selected based on literature data [22] and the pilot exposure analysis, which showed a marked increase in intracellular ROS production after 1 h treatment compared to untreated cells (Ctrl), without compromising cell viability. The positive control was incubated with EGCG (10 μg/mL) and 30 mM AAPH under the same conditions. Complete medium including DMSO (0.025% *v*/*v*) was used as a negative control. The incubation period was sufficient for the conversion of DCFH-DA to a highly fluorescent dichlorofluorescein (DCF). The fluorescence was measured using a fluorescent plate reader (Wallac 1420 multilabel counter Victor 3V, PerkinElmer, Shelton, CT, USA) at excitation and emission wavelengths of 485 and 535 nm, respectively. Data were expressed as relative fluorescence intensity, with the mean values represented as bars, from at least three independent experiments performed in triplicate (*n* = 9).

### 2.6. Wound Healing Assay

The cell scratch assay was used to assess the wound-healing potential of wine concentrates. The cells were seeded in a 96-well plate at 2 × 10^4^ cells/well in a final volume of 100 μL per well and incubated until the cells reached 100% confluence, with the medium changed after 24 h. A scratch across a cell monolayer was made using a sterile 200-μL pipette tip. The cells were rinsed with PBS to remove detached cells. The pre-selected fields were observed using an inverted microscope at 10× objective magnification and photographed using brightfield mode. After that, the cells were incubated with 50 μg/mL of the samples for 24 h. The corresponding vehicle control contained an identical final concentration of DMSO as the treatment group (0.025% *v*/*v*). To obtain the same field during image acquisition, reference points were marked on the outer bottom of the dish with a fine permanent marker. Following the incubation period of 24 h, new photographs were made in order to compare the wound area before and after the treatment, using the software ImageJ version 1.53e. The wound area was quantitatively assessed using the ImageJ “Wound Healing” plugin, which measures the reduction in the scratch area, and expressed as the percentage of closure relative to the initial scratch area. The mean values were represented as bars, from at least four independent experiments performed in triplicate (*n* = 12).

### 2.7. Quantitative Polymerase Chain Reaction (qPCR) Analysis

For gene expression studies, HaCaT cells were seeded in a 24-well plate at 2 × 10^5^ cells/well. On the following day, the cells were co-incubated with 50 μg/mL of the samples and 10 ng/mL of recombinant human tumor necrosis factor alpha (TNF-α, ab259410, Abcam, Cambridge, UK) for another 6 h. Then, the cells were rinsed with PBS and lysed in TRI reagent solution (Invitrogen, Thermo Fisher Scientific, Waltham, MA, USA). Total RNA was isolated and first-strand cDNA was synthesized from 700 ng of total RNA, using 0.5 μg of Oligo(dT) 12–18 primers, 250 μM of each dNTP, and 200 U of RevertAid reverse transcriptase. Real-time PCR was performed using the 7500 Real-Time PCR System (Applied Biosystems, Thermo Fisher Scientific, Waltham, MA, USA). The reaction mixture contained 1 μL of cDNA, 5 μL of 2× SYBR GREEN PCR Master Mix (Applied Biosystems, Thermo Fisher Scientific, Waltham, MA, USA), and specific forward and reverse primers in a final concentration of 0.5 μM for β-actin (*ACTB*), interleukin 1β (*IL1B*), IL-8 (*CXCL8*), and nuclear factor κB (*NFKB1*), and 0.1 μM for IL-6 (*IL6*). Reactions were run at 95 °C for 10 min, followed by 40 cycles of 15 s at 95 °C and 1 min at 60 °C. Expression levels of target genes were normalized to the housekeeping gene β-actin. Relative gene expression was calculated by the 2^−ΔΔCt^ method. The sequences of primers used in this study are given in Table 1. The experiment was performed four times in duplicate.

### 2.8. Enzyme-Linked Immunosorbent Assay (ELISA)

To evaluate the inflammatory response in HaCaT cells, concentrations of secreted IL-8 and TNF-α from cell culture supernatants were measured by ELISA. HaCaT cells were seeded in a 96-well plate at 2 × 10^4^ cells/well in a final volume of 100 μL per well. After 24 h, the cells were treated with wine concentrates (50 μg/mL) and co-incubated with recombinant human protein TNF-α (10 ng/mL) for another 6 h. Next, the cell medium was collected and used for the quantification of IL-8 and TNF-α using the Interleukin-8 ELISA Kit and the Human TNF-α ELISA Kit (Wuhan Fine Biotech Co., Ltd., Wuhan, China). The assay was conducted following the manufacturer’s protocol, and absorbance was measured at 450 nm using a microplate reader (Wallac 1420 multilabel counter Victor 3V, PerkinElmer, Shelton, CT, USA).

### 2.9. Statistical Analysis

For biological assays, data were analyzed by one-way and two-way analysis of variance (ANOVA) with Dunnett’s multiple comparison post hoc test. The values were expressed as mean ± standard deviation, and the differences were considered statistically significant at *p* < 0.05. For statistical analysis, GraphPad Prism, version 8.4.3 (GraphPad Software, Inc., Boston, MA, USA) was used.

The variability of polyphenolic profile, as well as expressed biological activities (inhibition of tyrosinase, elastase, and collagenase activities, antioxidant activity, wound area reduction, and anti-inflammatory activity, i.e., IL-8 production), among examined wine concentrates was determined by using principal component analysis (PCA). The data analyzed were standardized to account for the different magnitudes; hence, the responses and parameters contribute equally to the data set variance and to the principal component calculation. Statistical analysis was carried out using the STATISTICA version 14.0.015. (TIBCO Software Inc., San Ramon, CA, USA).

## 3. Results and Discussion

### 3.1. Polyphenolic Composition of the Analyzed Wine Concentrates

The polyphenolic composition of the four wine concentrates was previously characterized and reported in our earlier studies [16,17,18]. To improve the readability of the manuscript and provide the phytochemical context for the biological investigations presented herein, these previously published data are summarized in Table 2. They are included solely to facilitate interpretation of the biological results and do not represent new experimental findings of the present study.

A previous study of *V. vinifera* Merlot, Tannat, and Syrah grape skins identified 41 polyphenolic compounds, showing that polyphenol composition changes significantly during ripening, with decreases in flavan-3-ols and procyanidins, and increases in anthocyanins and stilbenes [15]. Besides grape variety, the concentration of resveratrol in grape-derived products is influenced by the stage of processing and production, showing variations throughout the sequence from grapes to concentrated grape juice, wine material, and finally wine [23,24]. In the current study, the analyzed wine concentrates exhibited noticeable differences in their polyphenolic composition (Table 2). For example, W3 exhibited remarkably higher total polyphenol and tannin contents (78.47 ± 4.06 µg eq gallic acid/mg dw and 51.37 ± 4.07 µg eq catechin/mg dw, respectively), while W2 was characterized by the highest total flavonoid content (1.593 ± 0.064 µg eq quercetin/mg dw). In contrast, W1 contained the highest total anthocyanin level (0.844 ± 0.010 µg eq malvidin-3-*O*-glucoside/mg dw), whereas W4 showed the lowest contents of most analyzed polyphenolic groups. Similarly, notable differences in the polyphenolic profiles were observed among the analyzed wine concentrates, which were further confirmed by PCA, based on the contents of the analyzed polyphenols (Figure S1). Briefly, W3 and W4 showed relatively similar positioning on the scores plot, indicating similarities in their phenolic composition, while W2 was clearly separated along PC1, suggesting a distinct profile compared to the other samples. W1 was differentiated along PC2, which may reflect differences in the abundance of anthocyanins and specific phenolic compounds. However, given the limited number of analyzed wine concentrates (*n* = 4), these PCA results should be considered exploratory and interpreted with caution.

### 3.2. Enzyme Inhibition Studies

A process of skin aging can be initiated by various factors, and it is represented by wrinkle formation, dryness, and reduced skin elasticity and firmness [11]. Aged skin is more prone to damage and the development of skin disorders. Degradation of key ECM components, including elastin and collagen, is crucial for skin aging [25]. Activation of elastase and collagenase, ECM-degrading enzymes, can occur on different occasions, including prolonged exposure to UV radiation, pollution, and chemical agents [11]. Moreover, skin aging is also characterized by hyperpigmented spots that are a consequence of melanin overaccumulation. Tyrosinase catalyzes the rate-limiting step in melanin synthesis and uses L-tyrosine as a substrate. Due to the aromatic structure of natural polyphenols, these compounds can mimic the L-tyrosine structure and act as competitive inhibitors of tyrosinase [25].

Table 3 summarizes the results of the skin-related enzyme inhibition assays. Since the objective of this study was to compare the biological performance of wine concentrates as potential ingredients for topical cosmetic formulations, enzyme inhibition assays were performed at a single concentration selected to reflect their intended use rather than to determine concentration-dependent inhibitory potency. Consequently, the activities were expressed as equivalents of the corresponding reference inhibitors, instead of IC_50_ values. The results of tyrosinase inhibitory activity are presented as μg kojic acid equivalents per mg of dry weight, as kojic acid is a common tyrosinase inhibitor [25]. The results of elastase and collagenase inhibitory activity are presented as μg EGCG equivalents per mg of dry weight, knowing that EGCG is a potent elastase and collagenase inhibitor [9].

Sample W3 exhibited the broadest anti-aging potential, showing strong tyrosinase inhibition and the highest collagenase inhibitory activity, although its elastase inhibition was comparatively weaker (Table 3). This suggests that sample W3 may be particularly effective for combined anti-wrinkle and depigmenting applications, with a lesser contribution to improving skin elasticity. Sample W4 demonstrated a more targeted activity profile, displaying the strongest elastase inhibition and solid collagenase inhibition, but lower tyrosinase inhibition, indicating its primary relevance for improving skin elasticity and supporting anti-aging effects related to elastin preservation. Sample W2 showed a more balanced activity, with strong tyrosinase inhibition and moderate elastase inhibition, but relatively weak collagenase inhibition, suggesting a greater suitability for depigmenting applications with some anti-aging potential. In contrast, sample W1 exhibited the lowest overall bioactivity across all tested enzymes, indicating a limited functional potential in these assays.

The distinct enzyme inhibitory profiles of the wine concentrates may, at least partly, reflect differences in their polyphenolic composition (Table 2). W3, which exhibited the highest collagenase inhibitory activity, was characterized by the highest total polyphenol and tannin contents and particularly high gallic acid content. In contrast, W2, one of the most active samples against tyrosinase, contained the highest total flavonoid content and was rich in caffeic, *p*-coumaric, and syringic acids. Interestingly, W4 exhibited the strongest elastase inhibition despite its comparatively lower contents of most polyphenolic groups, suggesting that the observed activities cannot be attributed solely to total polyphenol levels but may depend on the specific phenolic composition and interactions among individual constituents.

Although direct quantitative comparison is not possible because previous studies commonly reported enzyme inhibitory activity as IC_50_ values, these studies support the ability of grape- and wine-derived polyphenolic preparations to inhibit enzymes relevant to skin aging [25,26]. For example, red wine lees extract (from the Tempranillo variety) exhibited strong inhibitory activity towards tyrosinase, elastase, and collagenase (IC_50_~0.2 mg/mL for all the enzymes tested) [25]. Pomaces from red and white grapes (Malbec and Torrontes varieties, respectively) differ significantly in tyrosinase inhibitory activity; Torrontes extract showed no effect on tyrosinase activity, whereas the Malbec variety exhibited high potential in inhibiting this enzyme (IC_50_~90 μg/mL). It suggests that different levels of flavonoids between these two varieties may account for the observed differences in their bioactivity, indicating that a pomace with higher flavonoid content could be used as a skin-whitening agent [26]. On the other hand, Wittenauer et al. [27] found that white grape pomace extract strongly inhibited elastase and collagenase activity, while isolated phenolic compounds, including gallic acid, catechin, EGCG, procyanidin B1, and procyanidin B2, exhibited moderate collagenase inhibition and negligible elastase inhibition. These results suggest that phenolic compounds in the extracts act synergistically, highlighting their potential to mitigate skin aging [27].

In general, a limitation of the enzyme inhibition studies is that activities were evaluated at a single concentration and expressed as reference inhibitor equivalents; therefore, direct comparison with studies reporting IC_50_ values is not possible. Future studies employing concentration–response experiments will be required to determine the inhibitory potency of the wine concentrates and enable direct comparison with other extracts.

### 3.3. Viability of Wine-Treated HaCaT Cells

**Figure 1:**
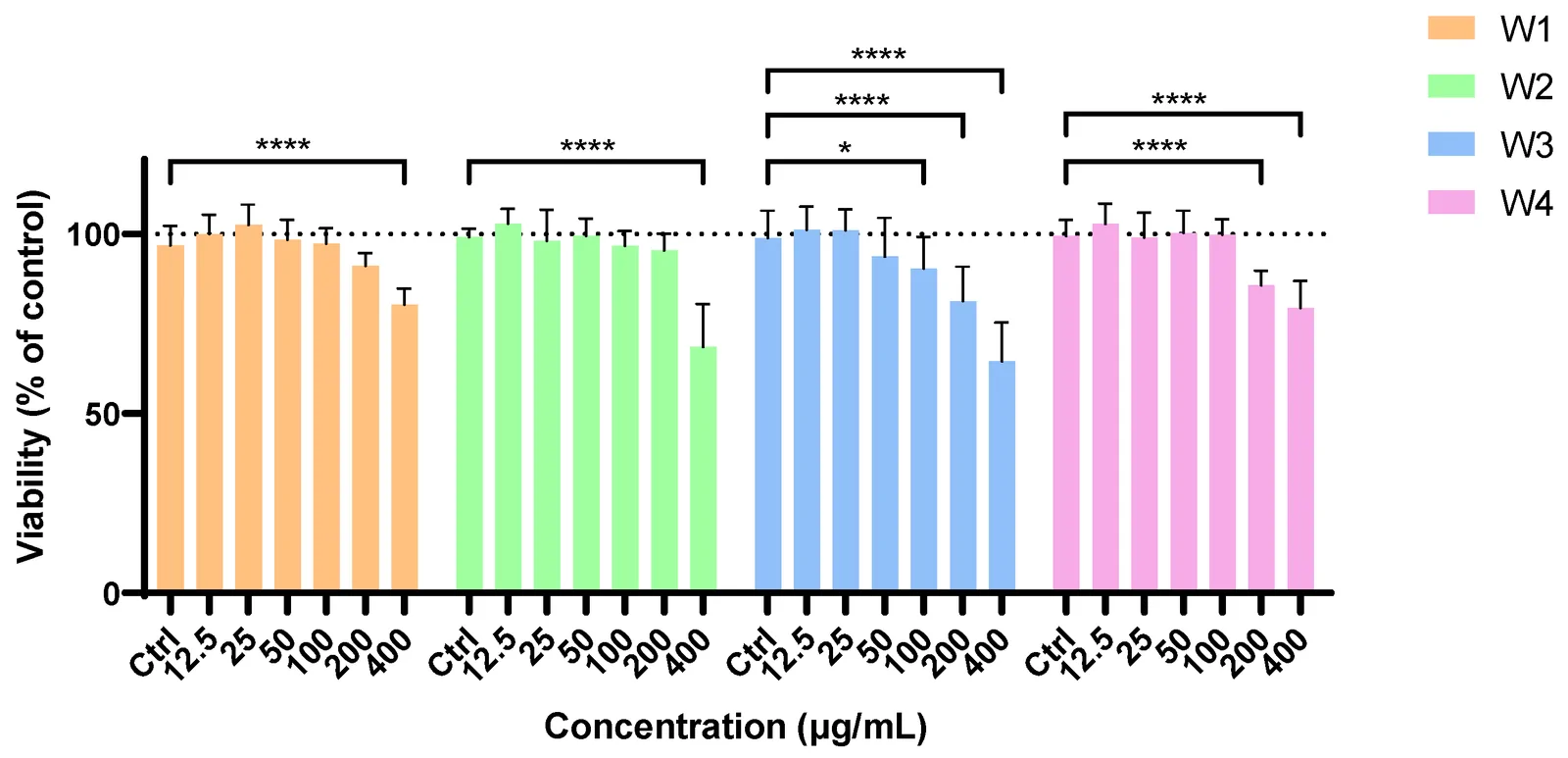
Viability of HaCaT cells after a 24 h incubation period with different concentrations of wine concentrates (12.5–400 μg/mL) compared to non-treated cells (vehicle control containing matching DMSO concentration), determined by the MTT assay. Data are expressed as mean ± standard deviation (* *p* < 0.05, **** *p* < 0.0001 vs. Ctrl) by two-way analysis of variance (ANOVA) with Dunnett’s multiple comparison post hoc test, *n* = 9. Abbreviations: W1: Serbian Cabernet Sauvignon concentrate; W2: North Macedonian Merlot concentrate; W3: Italian Merlot concentrate; W4: Serbian Merlot concentrate; Ctrl: control.

**Figure 2:**
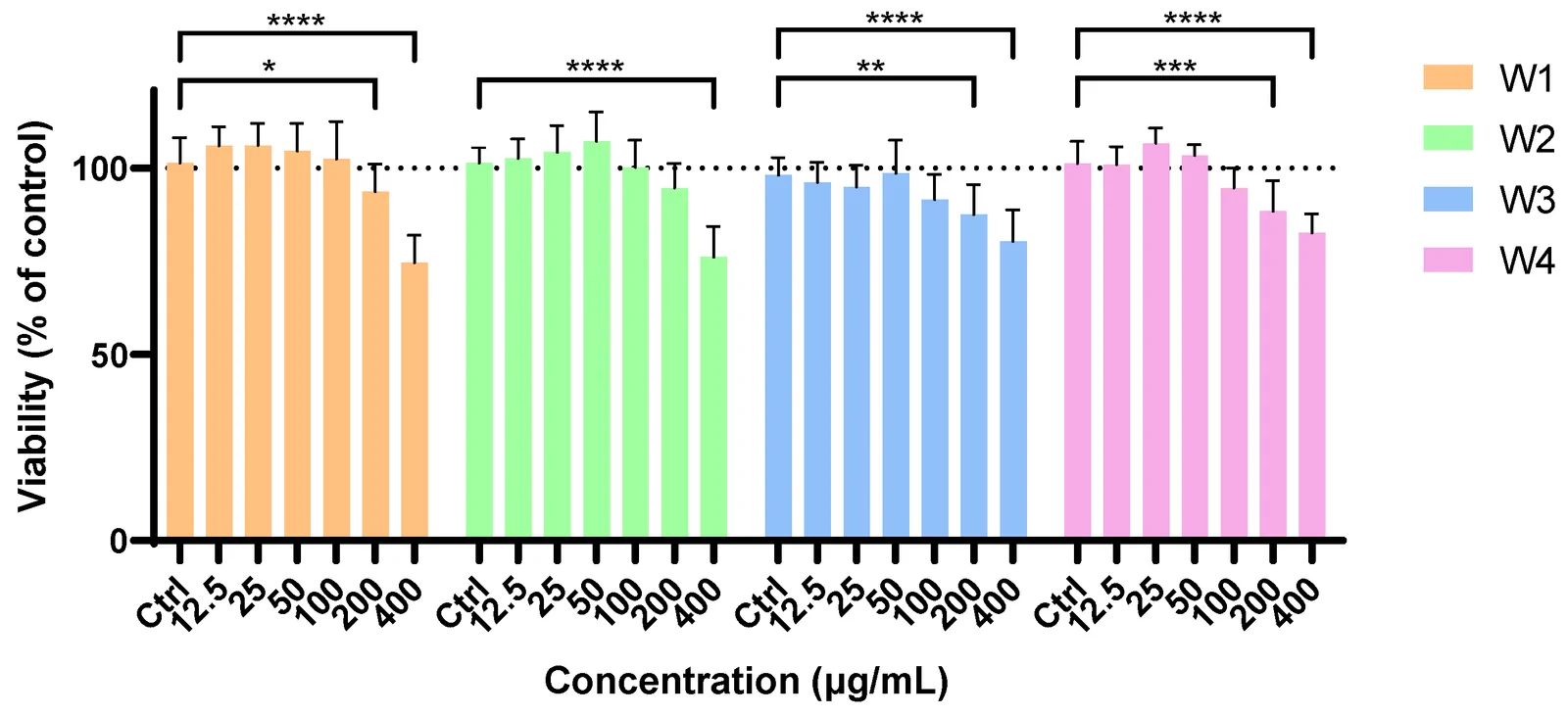
Viability of HaCaT cells after a 24 h incubation period with different concentrations of wine concentrates (12.5–400 μg/mL) compared to non-treated cells (vehicle control containing matching DMSO concentration), determined by CV assay. Data are expressed as mean ± standard deviation (* *p* < 0.05, ** *p* < 0.01, *** *p* < 0.001, **** *p* < 0.0001 vs. Ctrl) by two-way analysis of variance (ANOVA) with Dunnett’s multiple comparison post hoc test, *n* = 9. Abbreviations: W1: Serbian Cabernet Sauvignon concentrate; W2: North Macedonian Merlot concentrate; W3: Italian Merlot concentrate; W4: Serbian Merlot concentrate; Ctrl: control.

Cytotoxicity evaluation is a critical preliminary step in assessing the biological activity of tested samples, as it enables the identification of concentrations that do not adversely affect cell viability or metabolic function. Establishing non-cytotoxic exposure levels is essential to ensure that any effects observed in subsequent experiments can be attributed to specific biological mechanisms rather than to nonspecific cellular damage or loss of viability. In the present study, a non-toxic concentration was defined as one maintaining at least 90% cell viability compared to the untreated control. Cytotoxicity of wine concentrates was determined by MTT and CV assays, using the HaCaT cell line as an in vitro model (Figure 1 and Figure 2).

When the MTT assay was conducted, all examined wine concentrates showed no significant decrease in cell viability up to a concentration of 50 μg/mL after 24 h of exposure (Figure 1). Samples W1 and W2 appeared to be non-cytotoxic even at higher concentrations, reducing cell viability only at 400 μg/mL, while W3 exhibited cytotoxic effects in all concentrations above 50 μg/mL. The detected cytotoxicity of W3 is consistent with its phytochemical profile, as it contained the highest levels of total polyphenols and tannins among the tested samples. Polyphenols and tannins are known to exert pro-oxidant effects at elevated concentrations, leading to reduced cell viability. Therefore, the observed cytotoxicity of W3 is attributable to its high content of these compounds, which at lower concentrations may contribute to beneficial bioactivity, but at higher concentrations can compromise cell survival. Since the MTT assay has limitations in that it reflects the metabolic activity of cells rather than the number of viable cells, the CV assay was performed to confirm the results obtained by the MTT assay. This assay is used to determine the number of adherent cells, which correlates with cell viability. Results show similarity to the MTT assay, with the samples being cytotoxic in concentrations of 200 and 400 μg/mL (Figure 2). These findings are in agreement with a previous study showing that a grape polyphenolic extract (derived from grape pomace) did not induce cytotoxicity in HaCaT cells at concentrations up to 0.94 mg/mL [28]. Based on the results of the MTT and CV assays, all tested wine concentrates were considered non-cytotoxic at concentrations of 12.5, 25, and 50 μg/mL, and these concentrations were therefore selected for subsequent experiments. For the intracellular ROS assay, all three non-cytotoxic concentrations were evaluated to examine potential concentration-dependent antioxidant effects. In contrast, the highest non-cytotoxic concentration (50 μg/mL) was selected for the remaining mechanistic assays, as it was considered the most appropriate for evaluating the biological potential of the investigated wine concentrates under identical experimental conditions while maximizing the likelihood of detecting biological effects. The objective of these experiments was to compare the biological activities of the investigated wine concentrates rather than to establish concentration–response relationships.

### 3.4. Antioxidant Activity of Wine Concentrates

Overproduction and accumulation of ROS in cells lead to oxidative stress; it occurs when the endogenous antioxidant defense system fails to neutralize the excess ROS [13]. Skin is constantly exposed to external factors that induce oxidative stress; therefore, its protection from the damaging effects of ROS is crucial. Polyphenolic compounds are recognized for their antioxidant effects. Due to their aromatic structure and the presence of several hydroxyl groups, they are effective ROS scavengers, showing potential to prevent oxidative damage and premature skin aging [29].

**Figure 3:**
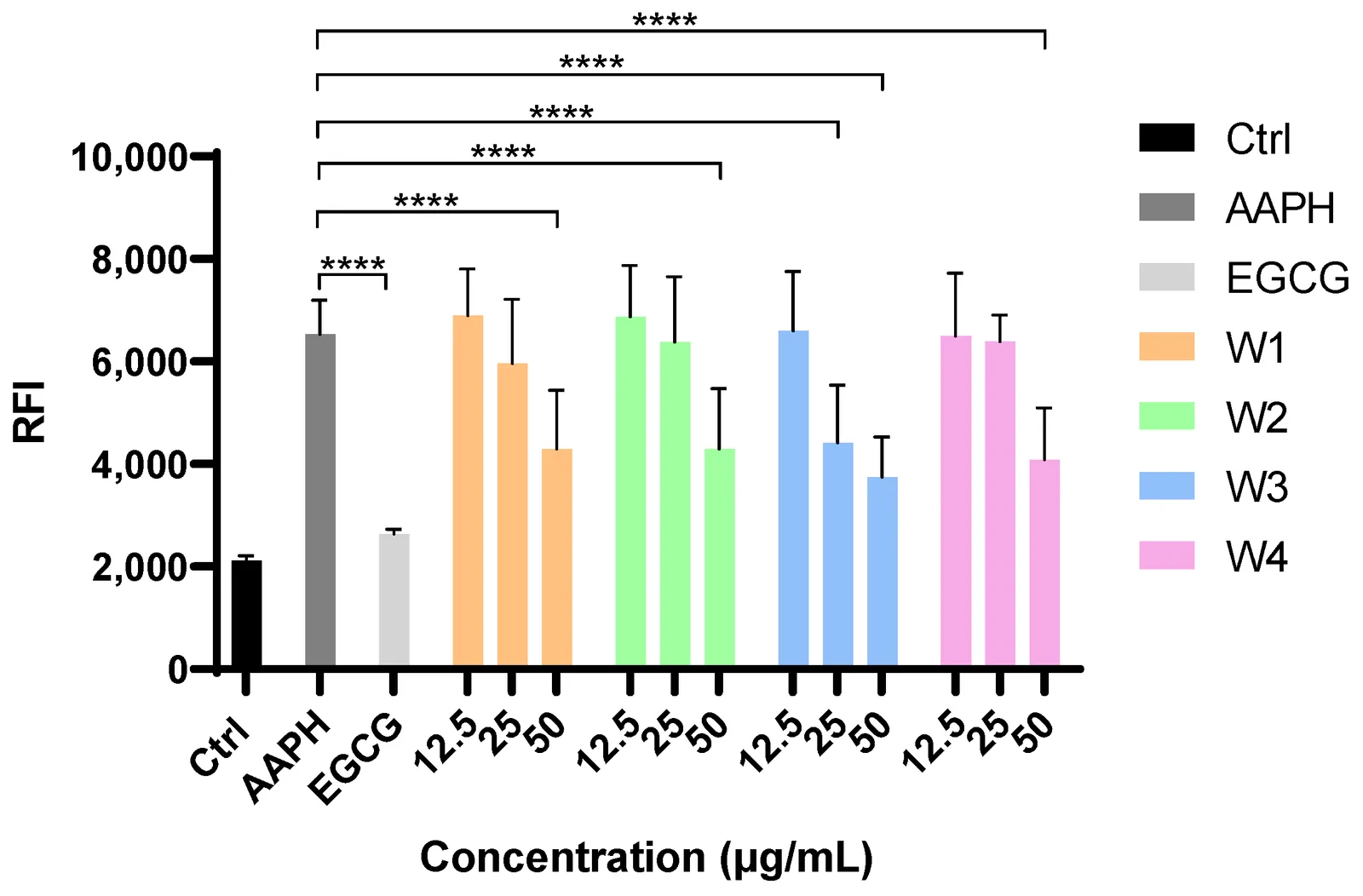
Effect of red wine concentrates (12.5–50 μg/mL) on intracellular reactive oxygen species levels in HaCaT cells, after 1 h co-incubation with 30 mM AAPH compared to AAPH-only treated cells, expressed as RFI and determined by DCFH-DA assay. Positive control was incubated with EGCG (10 μg/mL) and 30 mM AAPH under the same conditions. Data are expressed as mean ± standard deviation (**** *p* < 0.0001 vs. AAPH) by two-way analysis of variance (ANOVA) with Dunnett’s multiple comparison post hoc test, *n* = 9. Abbreviations: W1: Serbian Cabernet Sauvignon concentrate; W2: North Macedonian Merlot concentrate; W3: Italian Merlot concentrate; W4: Serbian Merlot concentrate; Ctrl: control; AAPH: 2,2′-azobisisobutyramidinium chloride; RFI: relative fluorescence intensity; EGCG: (-)-epigallocatechin gallate.

In this study, the antioxidant effects of red wine concentrates were assessed in a cellular model of oxidative stress by evaluating their ability to attenuate AAPH-induced intracellular ROS production in HaCaT cells at non-cytotoxic concentrations. Cells exposed to 30 mM AAPH for 1 h exhibited approximately a three-fold increase in relative fluorescence intensity (RFI) compared with untreated cells, indicating elevated intracellular ROS levels (Figure 3). In contrast, co-treatment with 50 μg/mL of the concentrates significantly reduced RFI relative to AAPH-treated cells, demonstrating their protective effect against AAPH-induced oxidative stress. This reduction was comparable to that achieved with EGCG (10 μg/mL), used as a positive reference antioxidant. It should be considered that red wine concentrates represent complex mixtures of multiple bioactive compounds, in contrast to pure EGCG. Therefore, their observed activity reflects a moderate cellular antioxidant effect within this oxidative stress model, with a measurable protective capacity that was achieved at a higher concentration compared with the reference antioxidant EGCG. Sample W3 showed the most pronounced protective effects among the tested concentrates, and significantly reduced ROS production even at the concentration of 25 μg/mL. This activity of W3 may be related to its distinct polyphenolic composition, as this sample contained the highest total polyphenol and tannin levels and markedly higher gallic acid content than the other concentrates (Table 2). Nevertheless, considering the complex composition of wine concentrates, the observed antioxidant activity likely reflects the combined contribution and interactions of multiple phenolic constituents rather than the effect of a single compound.

Similarly, Matos et al. [25] investigated the potential of red wine lees and grape pomace extracts to reduce intracellular ROS production in keratinocytes and fibroblasts. The study included pre-treatment with extracts, as well as a co-incubation of extracts and ROS inducer, *tert*-butyl hydroperoxide (TBHP). The pre-treatment demonstrates the preventive approach, while co-incubation represents a possible therapeutic strategy. Their results showed better antioxidant activity in the co-incubation setup. A possible explanation is that phenolic compounds are unable to permeate the cell membrane, and their activity can be observed only when present simultaneously with a stressor [25]. Conversely, Tempranillo grape pomace extract showed great antioxidant activity in pre-treated HaCaT cells. Namely, when the extract was incorporated in transfersomes, antioxidant activity was even stronger, indicating better absorption and bioavailability of the incorporated extract compounds [30].

Many polyphenolic compounds, present in grapes and wines, have shown antioxidant activity in HaCaT cells. Resveratrol [31] and 3,4-dihydroxybenzoic acid [32] inhibited ROS production in HaCaT cells exposed to UVB irradiation and H_2_O_2_, while catechin, procyanidin B2 [33], EGCG, and phosphatidylcholine-encapsulated EGCG [34], quercetin, and hyperoside [35] inhibited UVB-induced ROS production. Gallic acid inhibited TNF-α/IFN-γ-induced ROS [36], whereas myricetin inhibited only TNF-α-induced ROS production [37]. Previous studies demonstrated high concentrations of gallic acid and catechin in the tested samples [17,18], indicating that these compounds may account for the antioxidant potential of wine concentrates observed in this study.

### 3.5. Wound Healing Potential of Wine Concentrates

A wound represents an injury to the skin that is characterized by a loss of skin integrity due to a mechanical, chemical, or physiological agent [38,39]. Wound healing is triggered when the structural and functional integrity of the skin is disrupted. It is a four-stage process that includes hemostasis, inflammation, proliferation, and remodeling. A purpose of the wound healing process is the restoration of skin barrier function to prevent further damage or infection [39].

**Figure 4:**
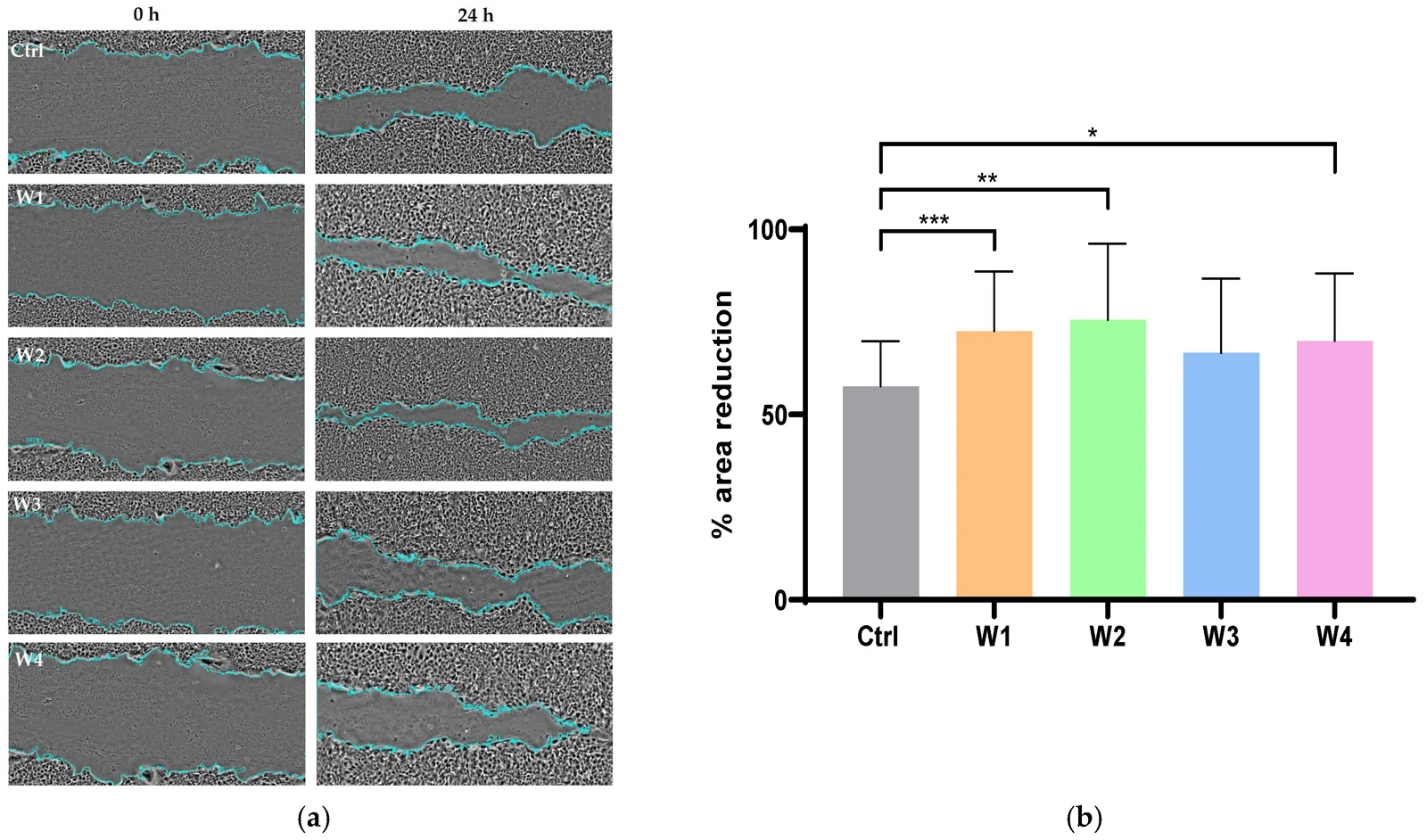
Effect of wine concentrates (50 μg/mL) on HaCaT cell migration after a 24 h incubation period compared to non-treated cells (Ctrl), determined by in vitro wound scratch assay. (**a**) Representative images of the wound scratch assay in treated keratinocytes, (**b**) Quantification of wound area reduction percentage of wound scratch assay. Data are expressed as mean ± standard deviation (* *p* < 0.05, ** *p* < 0.01, *** *p* < 0.001 vs. Ctrl) by one-way analysis of variance (ANOVA) with Dunnett’s multiple comparison post hoc test, *n* = 12. Abbreviations: W1: Serbian Cabernet Sauvignon concentrate; W2: North Macedonian Merlot concentrate; W3: Italian Merlot concentrate; W4: Serbian Merlot concentrate; Ctrl: control.

In order to examine the potential beneficial effects of wine concentrate samples on wound healing, a cell scratch assay was performed. A “wound” was created with a pipette tip, by making a linear scratch across a cell monolayer. The cells were incubated with 50 μg/mL of the samples, and cell migration was observed over 24 h. Figure 4a,b shows that all samples, except for sample W3, significantly stimulated cell migration, with sample W2 showing the highest rate of wound closure compared to non-treated cells (~75.5% vs. ~57.6% area reduction, respectively), highlighting its potential to promote tissue repair. The wound healing assay was performed using the highest non-cytotoxic concentration of the wine concentrates (50 μg/mL), which was selected based on the results of both the MTT and CV assays. Since this concentration did not alter the number of viable adherent cells compared with the control, the observed differences in scratch closure are unlikely to result from treatment-induced changes in cell viability or cell number, although a contribution of cell proliferation cannot be completely excluded.

Comparably, other studies showed that grape or wine extracts, as well as their main components, stimulated HaCaT cell migration. Specifically, grape seed extract obtained from the Pecorello variety stimulated wound healing in a dose-dependent manner [40]. Moreover, anthocyanin-rich wine extract, as well as isolated anthocyanins, cyanidin-3-glucoside, and malvidin-3-glucoside [14], along with gentisic acid [41] and quercetin [42], accelerated wound healing. Considering the significantly lower concentration of malvidin-3-*O*-glucoside in W3 compared to the other tested samples (Table 2), the observed results of wound healing potential are rather expected based on previous findings and correlate with the polyphenolic profile of the samples.

### 3.6. Modulation of TNF-α-Induced Inflammatory Responses by Wine Concentrates

Besides their barrier function, keratinocytes also exhibit immunological properties. In homeostasis, the production of inflammatory mediators by keratinocytes is kept at low levels. Various stimuli, including UV radiation, microorganisms, chemicals, and wound repair mechanisms, can promote the production of inflammatory mediators [43]. Cytokines like thymic stromal lymphopoietin (TSLP), TNF, and IL are important for the recruitment of immune cells in the skin [44]. TNF is commonly used as a pro-inflammatory agent in skin cell models [45]. In keratinocytes, it binds to TNF receptor 1 (TNFR1), triggering the activation of NF-κB and AP-1 signaling pathways, which upregulate the expression of numerous pro-inflammatory cytokines, chemokines, and adhesion molecules, including IL-1β, IL-6, and IL-8, thereby amplifying the inflammatory response [46].

**Figure 5:**
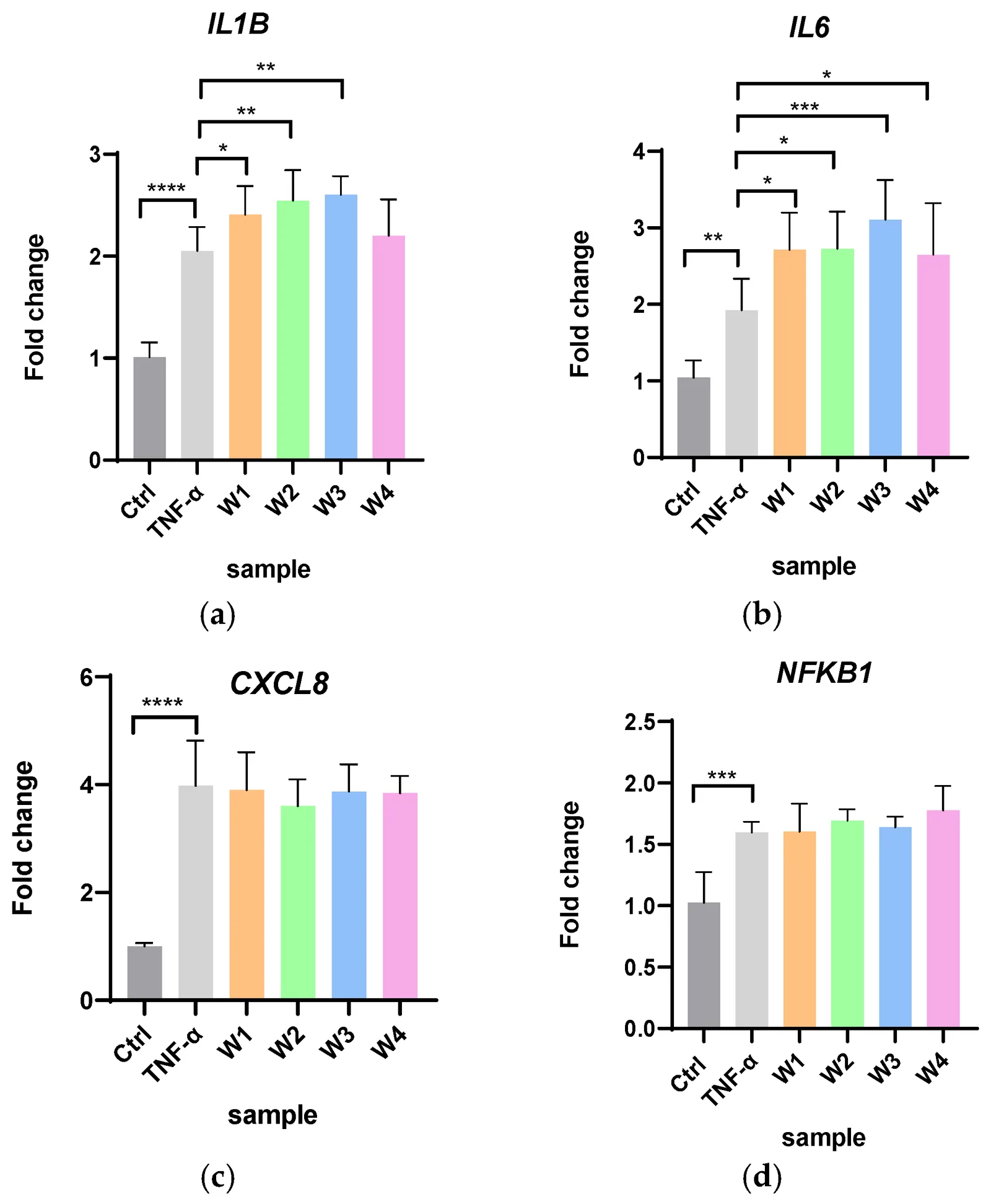
Effects of wine concentrates (50 μg/mL) on the levels of mRNA expression of inflammatory mediators: (**a**) IL-1β, (**b**) IL-6, (**c**) IL-8, and (**d**) NF-κB, after a 6 h incubation period with 10 ng/mL TNF-α. Data are expressed as mean ± standard deviation (* *p* < 0.05, ** *p* < 0.01, *** *p* < 0.001, **** *p* < 0.0001 vs. TNF-α-only-treated cells) by one-way analysis of variance (ANOVA) with Dunnett’s multiple comparison post hoc test, *n* = 8. Abbreviations: W1: Serbian Cabernet Sauvignon concentrate; W2: North Macedonian Merlot concentrate; W3: Italian Merlot concentrate; W4: Serbian Merlot concentrate; Ctrl: control; TNF-α: tumor necrosis factor alpha; IL: interleukin; NF-κB: nuclear factor κB.

For the assessment of inflammatory gene expression after treatment with wine concentrates, qPCR analysis was performed. The cells were exposed to 10 ng/mL of TNF-α and co-incubated with 50 μg/mL of the samples for 6 h before the analysis. The results show that TNF-α stimulates the mRNA expression of IL-1β, IL-6, IL-8, and NF-κB. Wine concentrates did not show any effect on IL-8 and NF-κB mRNA expression levels, while the expression of IL-1β was further increased when co-treated with samples W1–W3 compared with TNF-α-only-treated cells, and the same effects were observed in levels of mRNA for IL-6 in all the samples (Figure 5).

**Figure 6:**
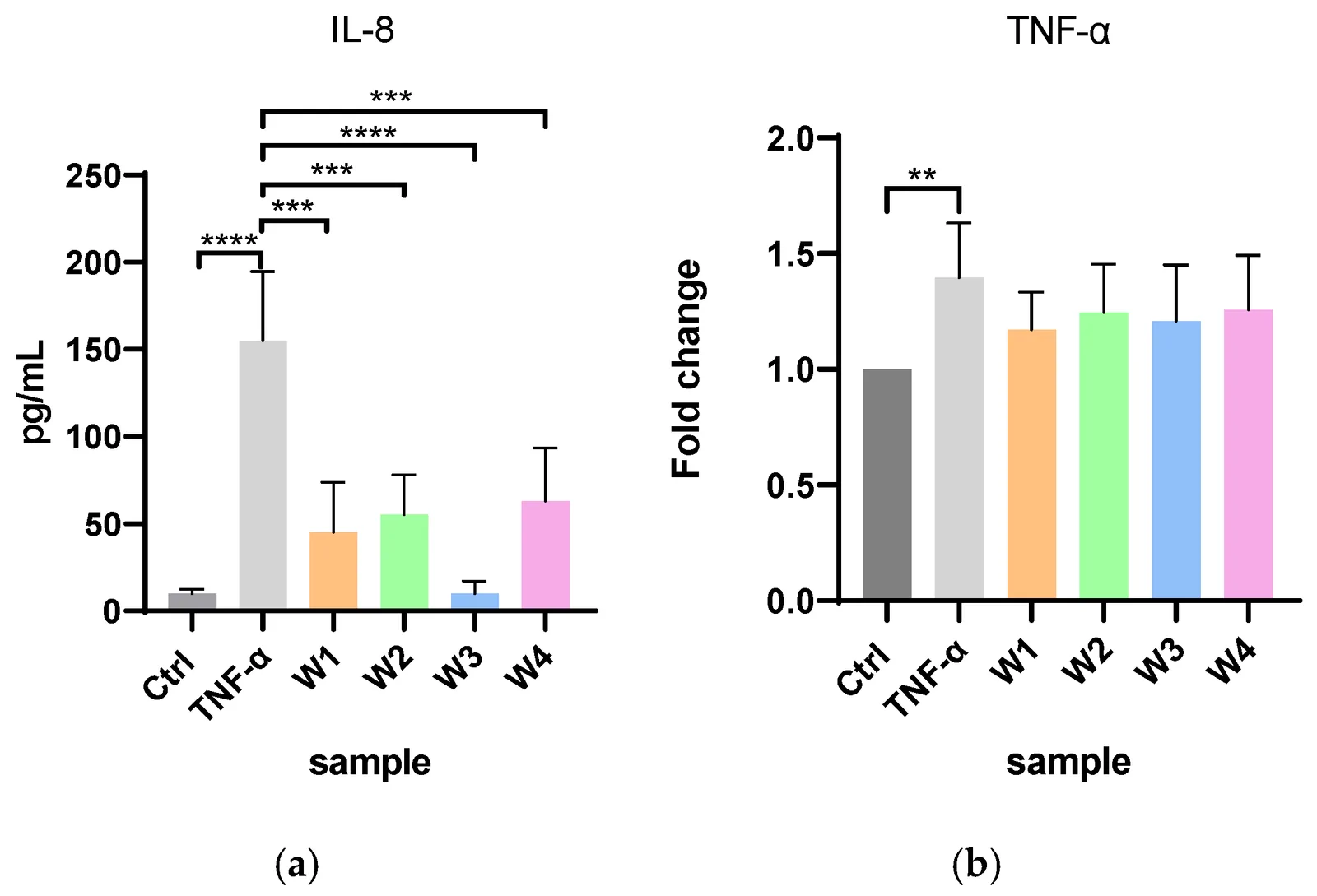
Effects of wine concentrates (50 μg/mL) on (**a**) IL-8 and (**b**) TNF-α levels after a 6 h incubation period with 10 ng/mL TNF-α. Data are expressed as mean ± standard deviation (** *p* < 0.01, *** *p* < 0.001, **** *p* < 0.0001 vs. TNF-α-only-treated cells) by one-way analysis of variance (ANOVA) with Dunnett’s multiple comparison post hoc test, *n* = 4. Abbreviations: W1: Serbian Cabernet Sauvignon concentrate; W2: North Macedonian Merlot concentrate; W3: Italian Merlot concentrate; W4: Serbian Merlot concentrate; Ctrl: control; TNF-α: tumor necrosis factor alpha; IL-8: interleukin-8.

The results of co-treatment with wine concentrates in TNF-α–stimulated cells suggest a direct interaction with TNF-α–induced signaling, indicating that the wine concentrates reshape inflammatory cascades. TNF-α signaling operates through key pathways, including the NF-κB and MAPK signaling pathways [47]. In this context, the selective enhancement of IL-6 and IL-1β mRNA expression, alongside the lack of effect on IL-8, indicates a targeted amplification of specific inflammatory pathways rather than a global increase in TNF-α responsiveness. These findings further support the presence of signaling divergence, whereby gene-specific modulation occurs, potentially through preferential activation of p38/AP-1 or inflammasome-related pathways driving IL-1β and IL-6 expression, without additional stimulation of NF-κB–dependent IL-8 [47,48,49]. Functionally, this pattern argues against the anti-inflammatory role of the concentrates under TNF-α stimulation and instead highlights their context-dependent immunomodulatory properties, skewing cytokine output toward a selective inflammatory profile. The observed increase in cytokine mRNA expression may also reflect a hormetic response induced by the polyphenol-rich concentrates. Although polyphenols are widely recognized for their anti-inflammatory properties, they can also act as mild stressors that transiently activate signaling pathways when used in higher concentrations [50]. This early adaptive response may enhance the transcription of inflammatory mediators, including interleukins, while simultaneously triggering cytoprotective mechanisms mediated by, e.g., Nrf2. Therefore, the increased cytokine gene expression observed in the present study may represent a transient adaptive stress response rather than a sustained pro-inflammatory effect. However, this interpretation should be considered with caution, as only a single concentration of wine concentrates (50 μg/mL) was evaluated, and the present experimental design does not allow confirmation of a true hormetic response, which requires a clear dose-dependent pattern and additional mechanistic validation. Further investigations involving multiple concentrations and analysis of relevant adaptive stress response pathways are required to determine whether hormesis contributes to the observed modulation of inflammatory mediators. To additionally evaluate the effects of wine concentrates on the modulation of TNF-α-induced inflammatory responses at the protein level, an ELISA was performed in supernatants of HaCaT cells co-treated with 50 μg/mL of wine concentrates and 10 ng/mL TNF-α. Figure 6 shows that the secretion of IL-8 was highly increased in TNF-α-treated cells, whereas the addition of the wine samples strongly inhibited this effect. Sample W3 exhibited the most significant effect, reducing IL-8 secretion to levels comparable to those of non-treated cells. On the other hand, TNF-α levels did not change significantly after treatment with wine concentrates. The combination of these results and results of qPCR analysis suggests that the regulatory mechanism may operate at the post-transcriptional level, as increased mRNA does not necessarily translate into increased cytokine secretion. Thus, post-transcriptional regulation may still lead to reduced protein levels despite the observed increase in mRNA expression. Our findings indicate that wine concentrates modulate TNF-α-induced inflammatory responses in a mediator-specific manner, rather than exerting a uniform anti-inflammatory effect. While the reduction in IL-8 secretion suggests a potential regulatory effect on inflammatory mediator release, the increased expression of selected cytokine transcripts highlights the complexity of cellular responses to bioactive compounds and warrants further mechanistic investigation.

Other studies showed that grape seed extract exhibited anti-inflammatory potential by inhibiting IL-1α and IL-6 [51], while grape leaf extract inhibited IL-8 secretion [52]. Conversely, Sittek et al. demonstrated that co-treatment with a wine extract stimulated secretion of IL-8 and inhibited secretion of IL-6 in HaCaT cells, where inflammation was induced by TNF-α [45]. The observed differences may be attributed to variations in the chemical composition of the samples, as well as to the activation of distinct signaling pathways involved in the biological response. It was previously reported that gallic acid [36] and rutin [53] strongly inhibited the secretion of IL-8 in HaCaT cells, indicating their potential contribution to the anti-inflammatory activity of the examined wine concentrates.

Although the present findings demonstrate the considerable biological potential of the red wine concentrates in keratinocyte-based models, the topical efficacy of these polyphenols may be limited by their physicochemical properties. Many polyphenols are highly hydrophilic, chemically unstable, and susceptible to oxidation, which may restrict their penetration through the *stratum corneum* and consequently reduce their bioavailability following topical application [54]. Therefore, the biological activities demonstrated in the present study should be interpreted as evidence of intrinsic bioactivity rather than direct confirmation of in vivo topical efficacy. The identification of the most biologically active concentrate in this work provides a rational basis for the next phase of research, which will focus on developing liposomal formulations as delivery systems capable of encapsulating both the hydrophilic and lipophilic constituents of the wine concentrate. Such formulations may improve polyphenol stability, protect them from degradation, provide controlled release, enhance skin retention and penetration, and ultimately increase their therapeutic potential for topical skin applications [55]. Future studies will therefore include physicochemical characterization of the liposomes containing wine concentrate, determination of encapsulation efficiency, in vitro release kinetics in simulated skin conditions (Franz diffusion cell studies comparing free and liposome-encapsulated wine concentrate), and evaluation of whether the formulation improves the biological performance of the selected concentrate.

### 3.7. Principal Component Analysis (PCA) of Samples and Their Biological Activity

**Figure 7:**
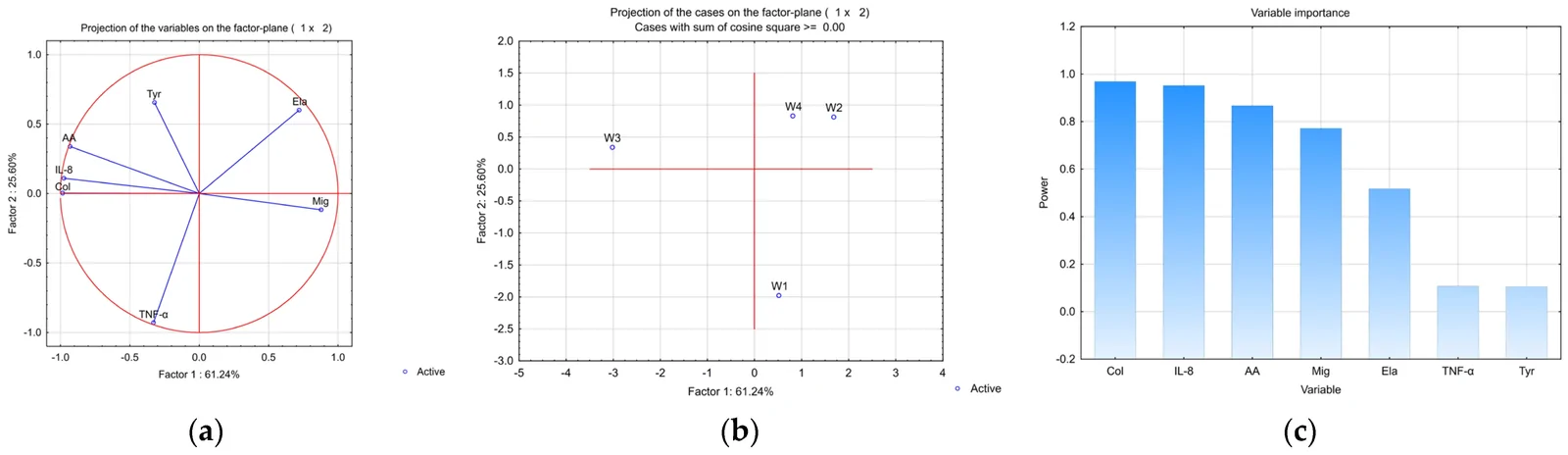
PCA of biological activity of analyzed wine concentrates: (**a**) Distribution of variables on the loadings plot; (**b**) Distribution of wine samples on the scores plot; (**c**) Variable importance. Abbreviations: (**a**,**c**) Tyr: inhibition of tyrosinase; Ela: inhibition of elastase; Col: inhibition of collagenase; AA: antioxidant activity (only the antioxidant activity determined at 50 μg/mL was included in the PCA in order to ensure comparability with the other cell-based biological assays, which were also performed at the same concentration); Mig: wound area reduction; IL-8: IL-8 production; TNF-α: TNF-α production; (**b**) W1: Serbian Cabernet Sauvignon concentrate; W2: North Macedonian Merlot concentrate; W3: Italian Merlot concentrate; W4: Serbian Merlot concentrate.

To explore the relationships between wine varieties and their biological activities, PCA was performed. PCA of expressed biological activities (Figure 7, Table S1) showed a clear differentiation among the analyzed wine concentrates, with the first two components explaining 86.84% of the total variance (PC1: 61.24%; PC2: 25.60%). Collagenase inhibition, IL-8 production, and antioxidant activity were mainly associated with the negative side of PC1, while elastase inhibition and wound area reduction were positioned on the positive side of PC1. The scores plot indicated distinct biological profiles of the wine concentrates, with W3 associated with antioxidant activity, collagenase inhibition, and IL-8 modulation, whereas W2 and W4 were more related to elastase inhibition and wound healing activity. W1 was separated along PC2 and showed a modest association with TNF-α modulation, likely due to its slightly higher activity compared to the other samples. Variable importance analysis identified collagenase inhibition, IL-8 production, and antioxidant activity as the main contributors to the discrimination of the examined samples.

Although the limited number of analyzed samples does not allow firm conclusions regarding varietal or geographical influences and should be interpreted with caution, the PCA suggested certain similarities between the North Macedonian and Serbian Merlot concentrates (W2 and W4, respectively), while the Italian Merlot (W3) and the Cabernet Sauvignon concentrate (W1) exhibited more distinct biological profiles. A previous study comparing monovarietal and blended wines from *V. vinifera* Cabernet Sauvignon and Merlot demonstrated that blending alters the polyphenolic composition and influences antioxidant, anti-inflammatory, and enzyme-inhibitory activities of the final product [16]. Although all examined wines exhibited notable biological activity, the study showed that the bioactivity of each blend is unique and cannot be directly predicted from their polyphenol content alone.

In general, although PCA revealed distinct clustering patterns among the analyzed wine concentrates, the small number of samples (*n* = 4) limits the statistical robustness of the analysis. Therefore, the observed groupings should be considered exploratory and hypothesis-generating rather than evidence of definitive biological or structure–activity relationships. Future studies including a larger number of wine samples are needed to validate these trends.

## 4. Conclusions

This study found that the concentrates of European wines exhibited various skin-beneficial effects. Although tested wine concentrates demonstrated enzyme-inhibitory activity, the observed differences in inhibitory profile suggest that the choice of the concentrate should be based on the targeted biological effect. Furthermore, the samples exhibited protective effects against oxidative stress and inhibited IL-8 secretion, with the Italian Merlot showing the most pronounced protective effects in human keratinocytes. On the other hand, its effect on wound healing was negligible, while other varieties highly stimulated cell migration. Examined wine concentrates show significant potential as natural ingredients for pharmaceutical, dermo-cosmetic, and cosmetic formulations aimed at preventing oxidative stress- and inflammation-induced skin damage. Evaluating the effects on more complex skin models will give a deeper perspective on the potential use of these concentrates in treating skin conditions. Additionally, future studies will focus on the development of liposomal formulations of the selected red wine concentrate to improve the stability, skin bioavailability, and dermal delivery of its polyphenolic constituents, followed by physicochemical characterization and evaluation of their release in simulated skin conditions in a Franz diffusion cell, and skin permeation properties.

## Figures and Tables

**Table 1:** Primer sequences used for qPCR.

Gene	Primer Sequence
*ACTB*	Forward: 5′-AGAGCTACGAGCTGCCTGAC-3′ Reverse: 5′-AGCACTGTGTTGGCGTACAG-3
*IL1B*	Forward: 5′-TCCCCAGCCCTTTTGTTGA-3′ Reverse: 5′-TTAGAACCAAATGTGGCCGTG-3′
*IL6*	Forward: 5′- GGTACATCCTCGACGGCATCT-3′ Reverse: 5′-GTGCCTCTTTGCTGCTTTCAC-3′
*CXCL8*	Forward: 5′-CTCTCTTGGCAGCCTTCCTGATT-3′ Reverse: 5′-AACTTCTCCACAACCCTCTGCAC-3′
*NFKB1*	Forward: 5′-GCAGCACTACTTCTTGACCACC-3′ Reverse: 5′-TCTGCTCCTGAGCATTGACGTC-3′

**Table 2:** Polyphenolic composition ^1^ of the analyzed wine concentrates.

Sample	W1 *	W2 [18]	W3 [18]	W4 [17]
Total polyphenols (µg eq gallic acid/mg dw)	65.63 ± 1.52 ^b^	52.18 ± 4.98 ^c^	78.47 ± 4.06 ^a^	50.47 ± 3.41 ^c^
Total flavonoids (µg eq quercetin/mg dw)	1.056 ± 0.101 ^b^	1.593 ± 0.064 ^a^	1.009 ± 0.079 ^b^	0.873 ± 0.076 ^c^
Total tannins (µg eq catechin/mg dw)	24.12 ± 1.46 ^b^	15.71 ± 1.55 ^c^	51.37 ± 4.07 ^a^	19.69 ± 1.51 ^c^
Total anthocyanins (µg eq M-3-G/mg dw)	0.844 ± 0.010 ^a^	0.641 ± 0.042 ^b^	0.650 ± 0.048 ^b^	0.430 ± 0.020 ^c^
Phenolic acids (mg/g dw)				
Gallic acid	1.405 ± 0.088 ^c^	0.501 ± 0.042 ^d^	4.327 ± 0.003 ^a^	1.983 ± 0.036 ^b^
Caffeic acid	0.071 ± 0.006 ^d^	0.482 ± 0.019 ^a^	0.267 ± 0.001 ^b^	0.090 ± 0.003 ^c^
Chlorogenic acid	0.015 ± 0.001 ^b^	0.049 ± 0.001 ^a^	n.d.	n.d.
*p*-Coumaric acid	0.089 ± 0.006 ^b^	0.389 ± 0.020 ^a^	0.262 ± 0.002 ^a^	0.079 ± 0.001 ^b^
Syringic acid	0.122 ± 0.006 ^b^	0.280 ± 0.008 ^a^	0.064 ± 0.002 ^c^	0.027 ± 0.001 ^d^
Vanillic acid	0.238 ± 0.025 ^a^	0.222 ± 0.005 ^b^	0.243 ± 0.008 ^a^	n.d.
*trans*-Cinnamic acid	n.d.	n.d.	n.d.	0.027 ± 0.001 ^a^
Benzoic acid	0.021 ± 0.000 ^c^	0.056 ± 0.002 ^b^	0.056 ± 0.001 ^a^	0.057 ± 0.002 ^a^
*p*-Hydroxybenzoic acid	0.051 ± 0.005 ^a^	0.036 ± 0.001 ^b^	0.050 ± 0.001 ^a^	n.d.
Flavonoids (mg/g dw)				
Catechin	1.054 ± 0.088 ^a^	0.534 ± 0.028 ^b^	1.017 ± 0.006 ^a^	1.027 ± 0.017 ^a^
Naringenin	0.005 ± 0.000 ^c^	0.014 ± 0.000 ^a^	0.003 ± 0.000 ^d^	0.007 ± 0.000 ^b^
Quercetin	0.024 ± 0.000 ^c^	0.252 ± 0.005 ^a^	0.069 ± 0.002 ^b^	0.022 ± 0.001 ^c^
Rutin	0.158 ± 0.016 ^b^	0.136 ± 0.005 ^c^	0.290 ± 0.010 ^a^	n.d.
Hesperetin	n.d.	0.008 ± 0.000 ^a^	n.d.	n.d.
Kaempferol	0.014 ± 0.001 ^a^	0.019 ± 0.000 ^a^	0.012 ± 0.000 ^a^	n.d.
Stilbenes (mg/g dw)				
Resveratrol	0.055 ± 0.004 ^a^	0.059 ± 0.001 ^c^	0.045 ± 0.002 ^b^	0.053 ± 0.001 ^a^
Piceid	0.060 ± 0.003 ^a^	n.d.	n.d.	n.d.
Anthocyanins (mg/g dw)				
Delphinidin-3-*O*-glucoside	0.056 ± 0.004 ^a^	0.009 ± 0.000 ^c^	0.003 ± 0.000 ^c^	0.023 ± 0.000 ^b^
Cyanidin-3-*O*-glucoside	0.013 ± 0.001 ^a^	0.007 ± 0.000 ^c^	0.005 ± 0.000 ^c^	0.007 ± 0.001 ^b^
Malvidin-3-*O*-glucoside	0.639 ± 0.034 ^a^	0.511 ± 0.028 ^b^	0.004 ± 0.000 ^d^	0.183 ± 0.009 ^c^
Petunidin-3-*O*-glucoside	0.064 ± 0.004 ^a^	0.020 ± 0.001 ^b^	0.008 ± 0.001 ^c^	0.016 ± 0.001 ^b^
Peonidin-3-*O*-glucoside	0.038 ± 0.001 ^b^	0.013 ± 0.000 ^d^	0.083 ± 0.004 ^a^	0.018 ± 0.000 ^c^

^1^ Values are means ± standard deviation of three measurements. Means within each row with different letters (a–d) differ significantly (*p* ≤ 0.05). * W1—A manuscript including this sample (phenolic profile) has been prepared and is currently under review for publication. Abbreviations: W1: Serbian Cabernet Sauvignon concentrate; W2: North Macedonian Merlot concentrate; W3: Italian Merlot concentrate; W4: Serbian Merlot concentrate; eq: equivalents; dw: dry weight; M-3-G: malvidin-3-*O*-glucoside; n.d.: not detected.

**Table 3:** Anti-tyrosinase, anti-elastase, and anti-collagenase activities of red wine concentrates ^1^.

Sample	Tyrosinase (µg eq kojic Acid/mg dw)	Elastase (µg eq EGCG/mg dw)	Collagenase (µg eq EGCG/mg dw)
W1	5.317 ± 0.512 ^c^	4.091 ± 0.258 ^c^	135.5 ± 2.8 ^b,c^
W2	9.040 ± 0.412 ^a^	5.611 ± 0.158 ^b^	121.2 ± 11.5 ^c^
W3	8.885 ± 0.215 ^a^	3.732 ± 0.245 ^c^	166.5 ± 11.9 ^a^
W4	6.197 ± 0.389 ^b^	6.272 ± 0.577 ^a^	137.2 ± 2.4 ^b^

^1^ Values are means ± standard deviation of three measurements. Means within each column with different letters (a–c) differ significantly (*p* ≤ 0.05). Abbreviations: W1: Serbian Cabernet Sauvignon concentrate; W2: North Macedonian Merlot concentrate; W3: Italian Merlot concentrate; W4: Serbian Merlot concentrate; eq: equivalents; dw: dry weight; EGCG: (-)-epigallocatechin gallate.

## Data Availability

The raw data supporting the conclusions of this article will be made available by the authors on request.

## Supplementary Materials

The following supporting information can be downloaded at: https://www.mdpi.com/article/10.3390/pharmaceutics18081034/s1, Table S1: Principal component analysis (PCA) of the biological activity of the analyzed wine concentrates. Loadings of the variables for principal components, based on correlations; Figure S1: Principal component analysis (PCA) of phenolic acids, flavonoids, stilbenes, and anthocyanins content in analyzed wines: (a) Distribution of variables on the loading plot. (b) Distribution of wine samples on the scores plot.

## Abbreviations

The following abbreviations are used in this manuscript:

AAPH	2,2′-Azobisisobutyramidinium chloride
AP-1	Activator protein-1
Ctrl	Control
CV	Crystal violet
DCFH-DA	2′,7′-dichlorofluorescein diacetate
DMEM-F12	Dulbecco’s Modified Eagle’s Medium/Nutrient Mixture F-12 Ham
DMSO	Dimethyl sulfoxide
dNTP	Deoxynucleoside triphosphate
d.r.	Dry residue
dw	Dry weight
ECM	Extracellular matrix
EGCG	(-)-epigallocatechin gallate
ELISA	Enzyme-linked immunosorbent assay
FALGPA	N-[3-(2-furyl)acryloyl]-Leu-Gly-Pro-Ala
FBS	Fetal bovine serum
IFN-γ	Interferon gamma
IL	Interleukin
L-DOPA	3,4-dihydroxy-L-phenylalanine
MAPK	Mitogen-activated protein kinase
mRNA	Messenger ribonucleic acid
MTT	3-(4,5-dimethylthiazol-2-yl)-2,5-diphenyltetrazolium bromide
NF-κB	Nuclear factor κB
Nrf2	Nuclear factor erythroid 2-related factor 2
PBS	Phosphate-buffered saline
PCA	Principal component analysis
qPCR	Quantitative polymerase chain reaction
ROS	Reactive oxygen species
SANA	N-Succinyl-Ala-Ala-Ala-p-nitroanilide
TBHP	*Tert*-butyl hydroperoxide
TNF	Tumor necrosis factor
TNFR1	Tumor necrosis factor receptor 1
TSLP	Thymic stromal lymphopoietin
UV	Ultraviolet
W1–4	Wine concentrate 1–4
