# Supplementary material for: Comparative Investigation of Red Wine Concentrates as Multifunctional Skin-Protective Agents
Source: Pharmaceutics. 2026 Aug 20;18(8):1034. doi: 10.3390/pharmaceutics18081034 (PMC13516056; doi:10.3390/pharmaceutics18081034)
Supplement: Supplementary file 1 [file pharmaceutics-18-01034-s001.zip › pharmaceutics-4454045-supplementary.pdf]

# Supplementary material

*Principal component* ***analysis***

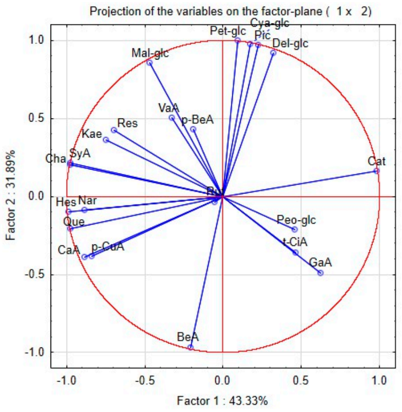


(**a**)

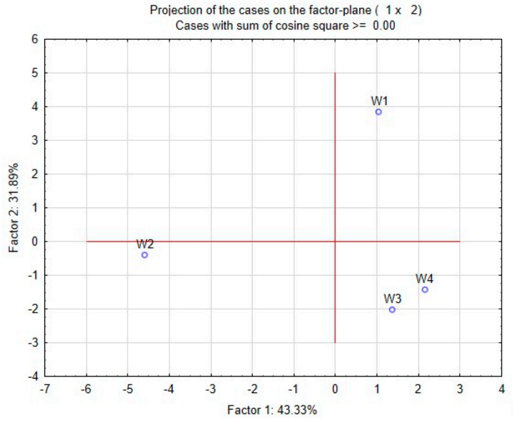


(**b**)

**Figure S1**. Principal component analysis (PCA) of phenolic acids, flavonoids, stilbenes, and anthocyanins content in analyzed wines: (a) Distribution of variables on the loading plot. (b) Distribution of wine samples on the scores plot.

Abbreviations: BeA: benzoic acid; CaA: caffeic acid; Cat: catechin; ChA: chlorogenic acid; Cya-glc: cyanidin-3-*O*-glucoside; Del-glc: delphinidin-3-*O*-glucoside; Hes: hesperetin; p-CuA: *p*-coumaric acid; p-BeA: *p*-hydroxybenzoic acid; GaA: gallic acid; Kae: kaempferol; Mal-glc: malvidin-3-*O*-glucoside; Nar: naringenin; Peo-glc: peonidin-3-*O*-glucoside; Pet: petunidin-3-*O*-glucoside; Pic: piceid; Que: quercetin; Res: resveratrol; Rut: rutin; SyA: syringic acid; t-CiA: *trans*-cinnamic acid; VaA: vanillic acid; W1: Serbian Cabernet Sauvignon concentrate; W2: North Macedonian Merlot concentrate; W3: Italian Merlot concentrate; W4: Serbian Merlot concentrate.

**Table S1**. Principal component analysis (PCA) of the biological activity of the analyzed wine concentrates. Loadings of the variables for principal components, based on correlations.

| Variable | Factor 1 | Factor 2 | Factor 3 |
|---|---|---|---|
| Tyr | -0.322 | 0.654 | -0.684 |
| Ela | 0.720 | 0.600 | 0.348 |
| Col | -0.984 | 0.003 | 0.178 |
| AA | -0.930 | 0.338 | 0.141 |
| Mig | 0.878 | -0.116 | -0.464 |
| IL-8 | -0.975 | 0.110 | -0.192 |
| TNF-$\alpha$ | -0.330 | -0.929 | -0.169 |
| Eigenvalue | 4.29 | 1.79 | 0.92 |
| Total variance (%) | 61.24 | 25.60 | 13.17 |
| Cumulative variance (%) | 61.24 | 86.83 | 100.00 |

Abbreviations: Tyr: inhibition of tyrosinase; Ela: inhibition of elastase; Col: inhibition of collagenase; AA: antioxidant activity (sample concentration 50 μg/mL); Mig: wound area reduction; IL-8: interleukin-8 production; TNF-$\alpha$: tumor necrosis factor-alpha production.
